# Data on the 21-Hydroxylase deficient CAH patients and the identification of known/novel mutations in CYP21A2 gene

**DOI:** 10.1016/j.dib.2016.12.013

**Published:** 2016-12-15

**Authors:** Ragini Khajuria, Rama Walia, Anil Bhansali, Rajendra Prasad

**Affiliations:** a Department of Biochemistry, Postgraduate Institute of Medical Education and Research, Chandigarh, India; b Department of Endocrinology, Postgraduate Institute of Medical Education and Research, Chandigarh, India

**Keywords:** CYP21A2 gene, Salt wasting, Simple virilizing, Non classical, Known mutations, Novel mutations

## Abstract

This article presents the dataset regarding spectrum of mutations in 21-Hydroxylase deficient CAH patients as described in “The spectrum of CYP21A2 mutations in Congenital Adrenal Hyperplasia in an Indian cohort” (R. Khajuria, R. Walia, A. Bhansali, R. Prasad, 2017) [1]. This dataset features about the CAH patients in the cohort, their classification into subtypes and finally screening the exon–intron boundaries of 21-Hydroxylase gene (CYP21A2) to detect common mutations, novel mutations along polymorphisms in the CYP21A2 gene. The specified large set of primers and the parameters for the mutation detection allow the identification and molecular characterization of CYP21A2 gene in the CAH patients.

**Specifications Table**

**Table:** 

Subject area	Biology
More specific subject area	Endocrinology, Molecular biology
Type of data	Text, Table, Graph, Figure
How data was acquired	Scatter diagram for 17-α-OHP, Primer sequences were checked using BLAST search
Data format	Analyzed
Experimental factors	DNA isolated from the blood of CAH patients
Experimental features	ELISA, Polymerase chain reaction
Data source location	Chandigarh, India
Data accessibility	Data is with this article and available at genbank via accession numbers: NCBI accession number-KF812549, NCBI accession number- KF534754, NCBI accession number- KF692099, NCBI accession number- KF447378

**Value of the data**

• The data provides the information about female to male ratio in the 21-Hydroxylase deficient CAH patients which could be compared to other studies.
• The data supports that level of 17-α-OHP in classical CAH patients is higher than non classical CAH patients as mentioned by other researchers and clinicians.
• The sequence of the primers mentioned would help other researchers to identify common and novel mutations in CYP21A2 gene.

## 1 Data

**Fig. 1:**
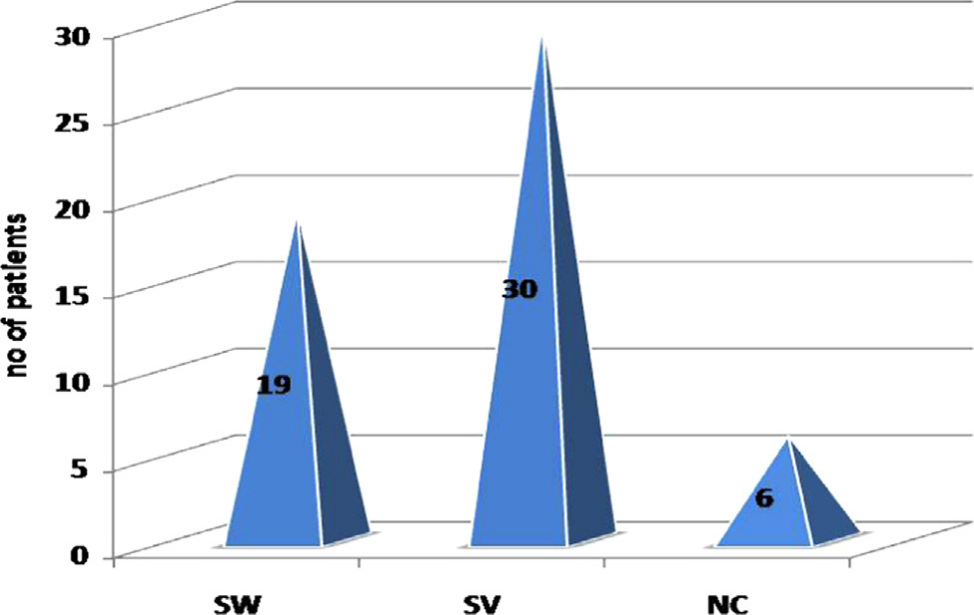
Number of patients in each sub-type of CAH.

**Fig. 2:**
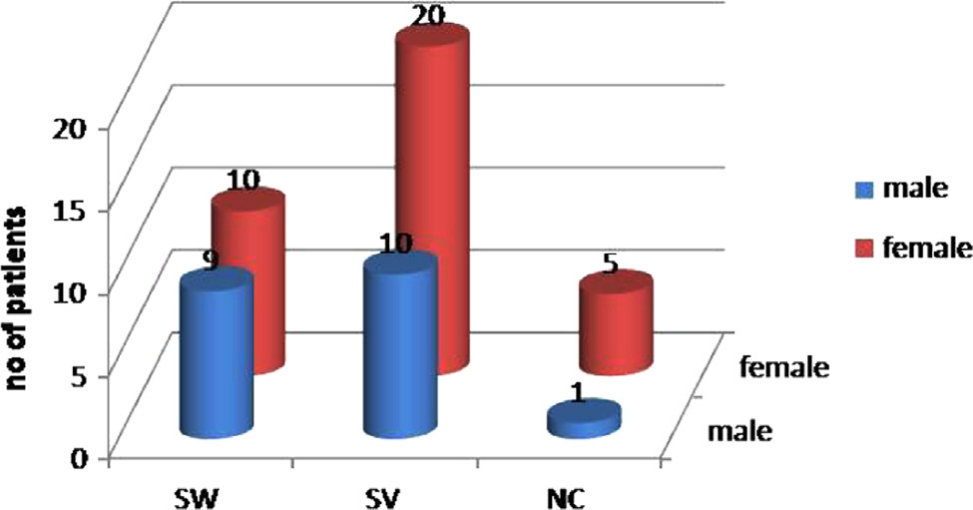
Number of patients in each sub-type of CAH along the male to female ratio in the three sub-types of CAH.

**Fig. 3:**
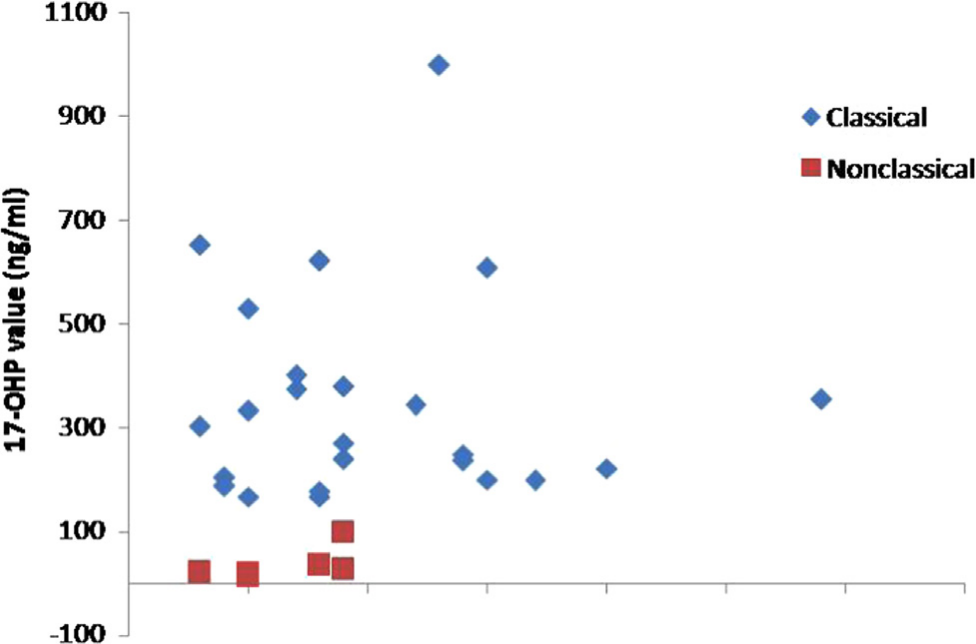
Scatter diagram representing the level of 17-α-OHP (ng/ml) in different categories of CAH (classical and non-classical). Levels of 17-α-OHP are higher in classical form of CAH as compared to non-classical form of CAH.

Congenital Adrenal Hyperplasia is an autosomal recessive disorder mainly caused by defects in 21-Hydroxylase gene (CYP21A2) which codes for 21-Hydroxylase enzyme [2]. Fig. 1, Fig. 2, Fig. 3 and Table 1 indicate ratio of patients (males and females) in classical (SW, SV) and non classical CAH and the associated level of 17-α-OHP which is the substrate of 21-Hydroxylase enzyme. The major disease-causing mutations in CYP21A2 (functional gene) are transferred from CYP21A1P (pseudogene) due to unequal crossing over during meiosis or apparent gene conversion events [3], macro or microconversion events [4]. Table 1, Table 2, Table 3, Table 4, Table 5 elucidate the age of the CAH patients in the classical and nonclassical CAH, the primer sequences which were used for detection of the common mutations, polymorphisms and novel mutations in the CYP21A2 gene. The novel mutations were detected at the frequency of 3%–5% when large cohorts were investigated [5] (Table 6).

## 2 Experimental design

### 2.1 Sample collection

The patients were categorized into 3 types viz., Salt Wasting (SW), Simple Virilizing (SV) and non classical (NC). CAH patients had varied age groups among the 3 types.

### 2.2 17-α-OHP measurement

17-α-OHP was measured in the serum samples of the CAH patients by enzyme-linked immune-sorbent assay (ELISA), based on the principle of competitive binding.

### 2.3 Identification of common mutations, polymorphisms and novel mutations

**Fig. 4:**
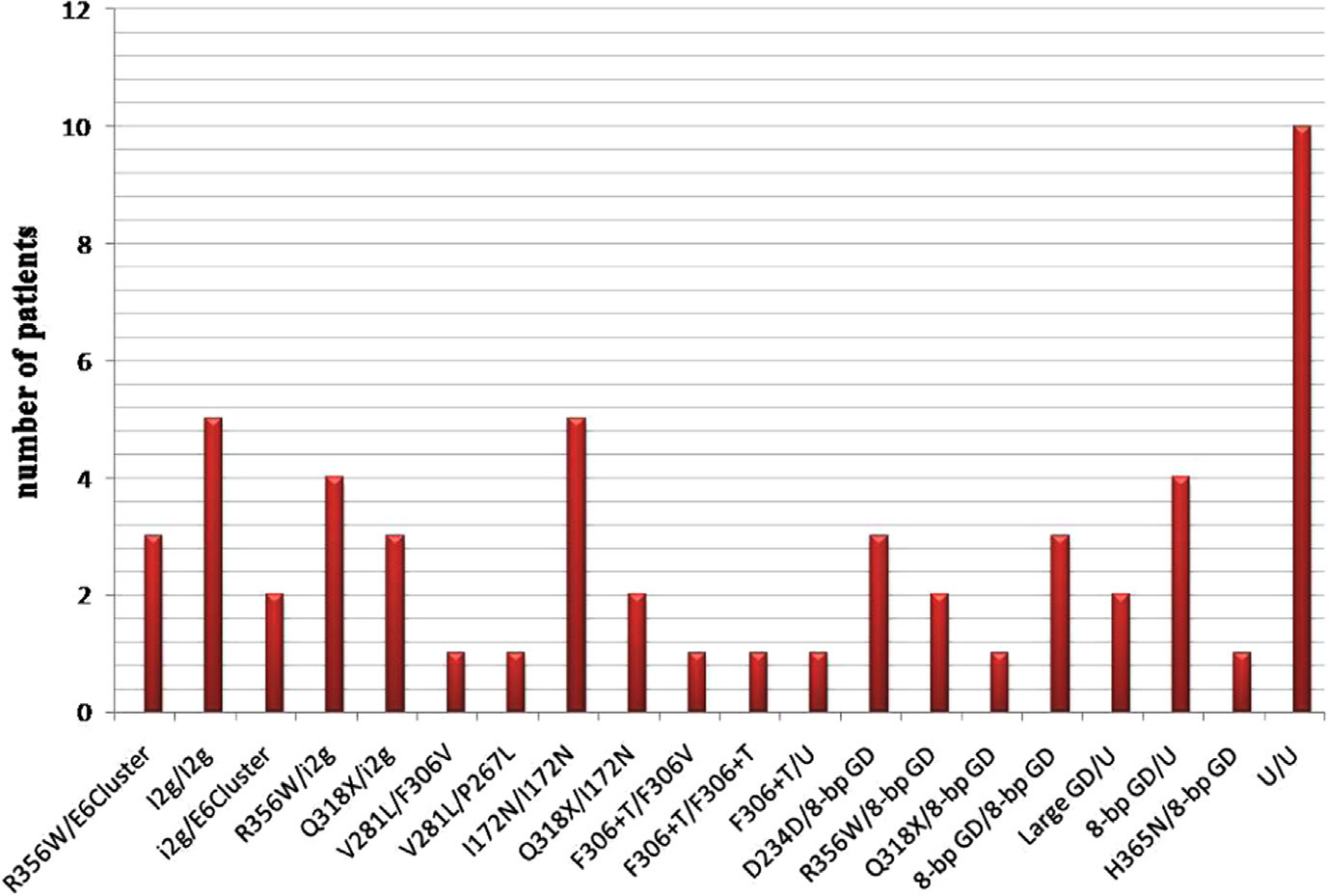
Type of genotype and their abundance in Indian CAH patients.

**Fig. 5:**
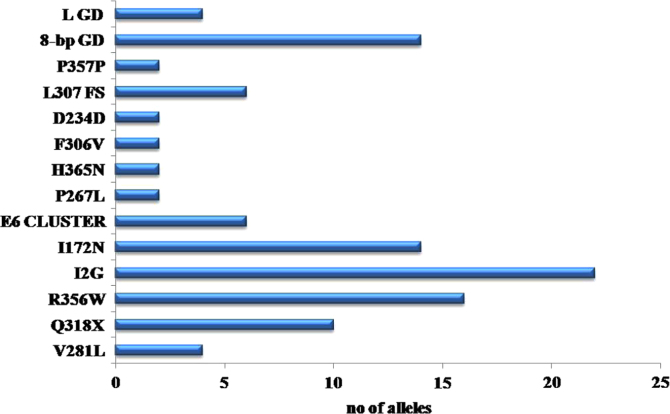
Bar diagram representing various mutations identified and the corresponding number of mutated alleles affected with each mutation.

**Fig. 6:**
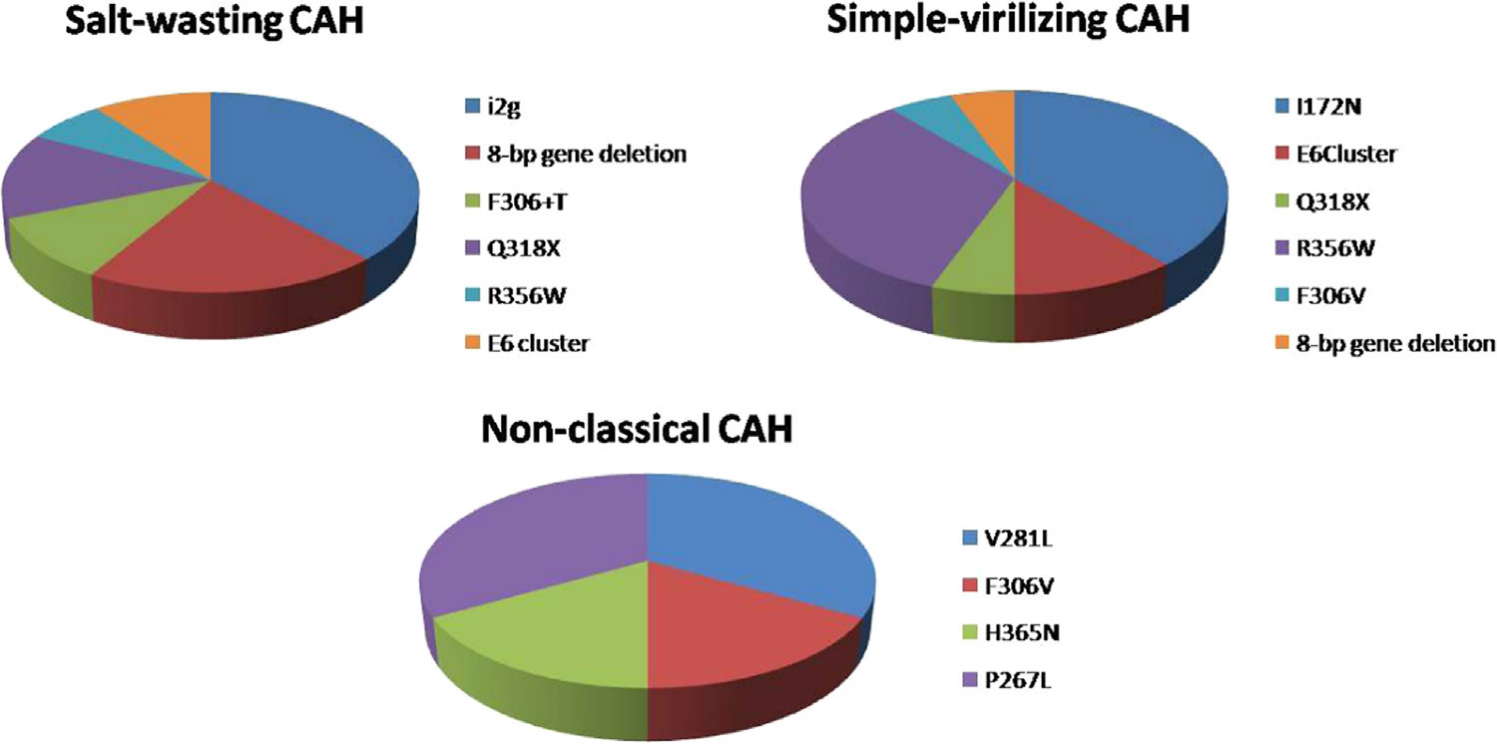
Mutations prevailing in different forms of CAH- Salt-Wasting, Simple-Virilizing and Non-Classical respectively in our population.

DNA was isolated by the standard protocols [6]. The common mutations, polymorphisms and the novel mutations were detected the 110 alleles [1]. Various mutations were present at different frequencies in our population [1]. The genotype of the patients and the affected no of alleles were detected in the present study (Figs. 4, 5). The prevalence of common mutations in 3 sub-types of CAH were also studied in present study (Fig. 6).

## Figures and Tables

**Table 1:** Range of age in CAH patients.

**Category of CAH**	**Minimum age (years)**	**Maximum age (years)**
Salt wasting	1 month	19
Simple virilizing	3.5	55
Non classical	17	24

**Table 2:** Oligonucleotides (primers) used to amplify the CYP21A2 gene including exon-intron boundaries. Column 1 is primer code, column 2 is primer sequence, column 3 is PCR product size (bp), Column 4 is mutation detected and column 5 is annealing temperatures(°C).

**1**	**2**	**3**	**4**	**5**
**P 1**	5′-TGC ATT TCC CTT CCT TGC TTC-3′	952	F1	63.2
**P 2**	5′-GCA GGG AGT AGT CTC CCA AGG- 3′^a^			
**P 3**	5′-CCT TGG GAG ACT ACT CCC TGC-3′	320	I172N E6 cluster	58.4
**P 4**	5′-AGG GGT TCG TAC GGG AGC AAT A-3′^a^	2070	F2	64.2
**P 5**	5′-CTG AGG TGC CAC TTA TAG CTC-3′^a^			
**P 6**	5′-AAG CTC CGG AGC CTC CAC CTC G-5′	148	P30L	51.5
**P 7**	5′-AGA TCA GCC TCT CAC CTT GC-3′^a^			
**P 8**	5′-TGG GGC ATC CCC AAT CCA GGT CCC-3′	156	i2g	62.0
**P 9**	5′-ACC AGC TTG TCT GCA GGA GGAT-3′^a^			
**P 10**	5′-TCT CCG AAG GTG AGG TAA CA T-3′^a^	320	I172N	58.4
**P 11b**	5′-AGC TGC ATC TCC ACG ATG GA-3′^a^	696	E6 cluster	60.6
			N allele	
**P 11a**	5′-TCA GCT GCT TCT CCT CGT TGT GG-3′^a^	696	E6 cluster	60.2
			M allele	
**P 12**	5′-GAT CAC ATC GTG GAG ATG CAG CTG-3′	781	V281L	71.0
**P 13**	5′TGG GCC GTG TGG TGC GGT GGG GCA A-3′^a^		Q318X	
**P 14**	5′CCA GAT TCA GCA GCG ACT G-3′	162	R356W	67.0
**P 15**	5′-TGG GGC AAG GCT AAG GGC ACA AC C-3′^a^			

a underlined primers are antisense primers.

**Table 3:** List of the mutations, PCR product size, the restriction enzyme used and the fragment size obtained after digestion for the detection of the common mutations.

**Mutation**	**PCR product**	**Restriction enzyme**	**Fragments produced after digestion if mutation present**	**Fragments produced after digestion if mutation absent**
P30L	148 bp	Bsh12361	148 bp	126 bp & 22 bp
**I172N**	320 bp	Nde 1	297 bp & 28 bp	320 bp
**V281L**	781 bp	Apa L1	686 bp & 95 bp	375 bp,311 bp & 95 bp
**i2g**	156 bp	Sau3A1	133 bp & 23 bp	156 bp
**Q318X**	781 bp	Pst 1	457 bp,204 bp & 120 bp	299 bp,204 bp,158 bp & 120 bp
**R356W**	163 bp	Eco521	162 bp	136 bp
**Gene Deletion**	210 bp	Taq 1	187 bp	210 bp & 187 bp

**Table 4:** List of the mutations, optimum temperature, time duration required for the detection of the known mutations.

**Mutation**	**Restriction enzyme**	**Temp.**	**Duration of incubation**
P30L	Bsh12361	37 °C	4 h
**I172N**	Nde 1	37 °C	4 h
**V281L**	Apa L1	37 °C	4 h
**i2g**	Sau3A1	37 °C	2 h
**Q318X**	Pst 1	37 °C	4 h
**R356W**	Eco521	37 °C	4 h
**Gene deletion**	Taq 1	65 °C	4 h

**Table 5:** Mutations or sequence variations, primers used for PCR and restriction enzymes used in detection of normal and mutant alleles.

**Poly-morphim**	**Primer**	**PCR product**	**Enzyme**	**Fragment Size (bp)**	
				**Normal allele**	**Mutant allele**
**S268T**	7-F TGCAGGAGAGCCTCGTGGCAGG 7-R ACGCACCTCAGGGTGGTGAAG	212 bp	Nco 1	–	–
					
**D183E**	5-F GGAGACAAGATCAAGGTGCCT	217 bp	–	212	146 and 66
	5-R CCAGGTCCTCACCCTGAGA				

**Table 6:** Oligonucleotides primers used for amplification of CYP21A2 gene exons.

**Exons**	**Primer Sequence 5′–3′**	**PCR product (bp)**	**Annealing temperature (°C)**
**Forward 1**	AGCGGATCCCCCGGTGGCCTC	216	63.0
**Reverse 1**	CCGTGGCCCAGCCTGCAGATG		
**Forward 2**	AGCTCTGAGGACTGATCTTGA	208	61.8
**Reverse 2**	CCGTGGCCCAGCCTGCAGATG		
**Forward 3**	AGCTCTGAGGACTGATCTTGA	226	66.4
**Reverse 3**	AGCAGCAGTTGGAGCCAGGTT		
**Forward 4**	GTACGATAGCACCTTCCTGTT	207	61.8
**Reverse 4**	GCTGAGTCTCCAACTCTGGTT		
**Forward 5**	TTGGGGTTCGCCCTGCCCGTA	217	68.6
**Reverse 5**	CAAAGCTTCATCACCCCCTCC		
**Forward 6**	AGGAGGGAGTTGACTTGGTGT	193	63.4
**Reverse 6**	CTGTTCCCATGTCCACAGTGC		
**Forward 7**	TGGGACAGAGGAAATATGCCA	212	65.5
**Reverse 7**	CCTTTACZCACCTCTCTCATG		
**Forward 8**	GGCTCCTATGTCACCTTGATG	227	62.1
**Reverse 8**	CAACCTCCATCCAGTGCCTAG		
**Forward 9**	GGTCAGCATCTGGACCCCAGG	212	66.9
**Reverse 9**	AGGTTGCAGTTCACTAGGCTG		
**Forward 10**	AGGTGCTAACCTGGATAACTG	303	59.8
**Reverse 10**	CACATACTGCATGTGAGAGTC		
