# Supplementary material for: Data on the 21-Hydroxylase deficient CAH patients and the identification of known/novel mutations in CYP21A2 gene
Source: Data Brief. 2016 Dec 15;10:406–12. doi: 10.1016/j.dib.2016.12.013 (PMC5330408; doi:10.1016/j.dib.2016.12.013)
Supplement: Supplementary file 1 — Supplementary Material [file mmc1.docx]

Conflict of interest

Authors have no conflict of interest
